# Appendicectomy and Clostridium Difficile Infection: Is There a Link?

**DOI:** 10.14740/jocmr1840w

**Published:** 2014-05-22

**Authors:** Charalampos Seretis, Fotios Seretis, Kolitha Goonetilleke

**Affiliations:** a Department of Colorectal Surgery, Good Hope Hospital, Heart of England NHS Foundation Trust, Birmingham, UK; b Medical School of Patras, Greece

**Keywords:** Appendix, Appendicectomy, Clostridium difficile, Pseudomembranous colitis, Surgery, Infection

## Abstract

Clostridium difficile infection (CDI) is a gradually emerging healthcare problem in the western world, occurring predominantly from the de-arrangement of the gut microbiota and the widespread use of antibiotics. Recently, it has been proposed that the presence or absence of the appendix could be a factor influencing the occurrence and/or the severity of CDI. We performed a review of the literature, aiming to identify and interpret in an accumulative way the results of the published clinical studies which addressed the issue of a possible association between prior appendicectomy and the features of CDI. A total of five suitable studies were retrieved, which were all conducted retrospectively. Although the results were conflicting regarding the impact of prior appendicectomy in the occurrence and relapse of CDI, it appears that the presence or absence of the appendix is not associated with the clinical severity of CDI. Based on the current evidence and considering the effects of the widespread use of antibiotics in the clinical practice, it appears that an *in situ* appendix does not have a definitive impact on the development and severity of CDI. Further observational studies are warranted to clarify any potential association.

## Introduction

Clostridium difficile infection (CDI) is considered to be a hospital-acquired infection, with its prevalence gradually increasing in the western world [1]. Although of a wide span in terms of clinical manifestations, CDI can lead to the establishment of fulminant, life-threatening colitis, which might even require surgical intervention [2]. However, even when presenting with mild diarrhea, CDI increases significantly the length of admissions, the morbidity and mortality rates and poses a constant threat to the function of the healthcare systems, both due to the increase of the hospitalization costs and due to the constant threat of intra-nosocomial spread of the infection [3-5].

There is undisputed evidence that the inappropriate and excessive use of antibiotics predisposes to the development of CDI, as it alters the normal gut flora, enabling the expansion of CD colonization in the colon [6, 7]. Despite the fact that the inappropriate use of antibiotics has for long been identified as a key-factor for CDI, very slow progress is being made in terms of prescribers’ education to tackle this phenomenon [8, 9]. Therefore, early recognition and prompt treatment is of cardinal importance in the case of CDI; from this point of view, the identification of groups of patients that could be in higher risk of developing CDI could be of particular value in our clinical practice.

Recently, a scientific debate has emerged regarding the possible association of prior appendicectomy and the occurrence of CDI. Although its exact function has not been clearly understood yet, the presence of gut-associated lymphoid tissue (GALT) in the appendix, along with its anatomic location close to the ileo-cecal valve, the functional barrier between the small and large intestine indicates that the appendix probably plays an important role in the regulation of the immune profile of the intestine [10]. More specifically, there is mounting evidence that the appendix, containing an extremely high concentration of gut microbiota, serves as an immunological reservoir of the lower gastrointestinal tract, enabling the re-inoculation of the colon with normal flora in cases of intestinal infections [11]. As a result, it would be reasonable to assume that patients who have previously undergone an appendicectomy would be more prone to the development of CDI in the absence of the immunological surveillance of the colon by an *in situ* and functioning appendix. In order to address this question of a possible association between previous appendicectomy and CDI and specifically whether an *in situ* appendix has a protective role against the occurrence, relapse and clinical severity of CDI, we performed a systematic review of the published English literature.

## Literature Search Methods

We performed a systematic review of the published clinical studies in PubMed which examined the association between prior appendicectomy and CDI. The keywords used in the search engine were the combinations of “clostridium difficile” or “pseudomembranous colitis” with the words “appendix”, “appendicectomy” and “appendectomy”. The literature search assessed for suitability all papers published in PubMed database until February 2014. We excluded from our review case reports and studies not published in English. The references of the retrieved papers were manually searched in order to identify further potentially relevant studies. The literature review was performed independently by two of the authors (CS and FS); any matters of controversy regarding the suitability of any studies were resolved between the authors. In cases where the full-text of any potentially relevant studies was not available, the corresponding authors were contacted to provide the full-length version of the article.

## Literature Review

Our literature search yielded a total of 89 records, with another eight additional papers being identified as potentially suitable through the manual search of the references. After the removal of the duplicate records (n = 43) and the papers not published in English (n = 11), a total of 43 papers were reviewed for inclusion in the review. From these, 28 were excluded as irrelevant to our review question and another 10 correseponed to case reports (n = 5) and literature reviews (n = 5), leaving five studies to be included in the review [12-16]. The main features and findings of each study are presented in a detailed manner in Supplementary 1 (www.jocmr.org).

In a general overview, all of the included studies were retrospective; our literature search did not identify any relevant prospective study. With respect to the main question our this review aims to address, which is whether the occurrence of CDI is more frequent in patients previously submitted to an appendicectomy, two of the studies reported higher rates of previous appendicectomies in the CDI negative compared to CDI positive groups of patients, although these differences were not statistically significant [12, 16]. In contrast with the latter, Clanton et al [15] found that there was a significantly higher rate of prior appendicectomy in their CDI positive group of patients compared to the national average. Moreover, the data were conflicting with respect to the possible association of appendicectomy with the recurrence of CDI. Im and co-workers [13] suggest that in their study group CDI recurrence rate for patients with an appendix was 18%, compared with 45% in those without an appendix, while Khanna et al [14] failed to demonstrate a similar association.

On the contrary, however, the results of the studies seem to be in accordance when referring to a possible association of the severity of CDI with the presence of an *in situ* appendix. More specifically, Ward et al, as well as Khanna et al and Clanton et al [12, 14, 15] did not find any correlation between the clinical severity of CDI or the presence of pathological features of ischemic changes in the removed colectomies’ specimens and a previous appendicectomy. Moreover, Khanna et al [14] suggested that the presence or absence of an intact appendix was not associated with adverse outcomes for the patients due to the clostridium difficile colitis.

## Conclusions

There is an increasing amount of evidence that the appendix has a significant role as a mediator of the lower gastrointestinal tract immune function, since the performance of appendicectomy has been recently suggested to prevent the development of ulcerative colitis and has been implemented as a treatment option for ulcerative proctitis [17, 18]. Taking into account the fact that CDI, especially in the face of widely inappropriate use of antibiotics in the clinical practice, has become a major healthcare problem, it would be tempting to assume that the presence or absence of an intact appendix could have an impact on the occurrence or natural course of CDI, especially under the notion that the appendix has evolved as a immuno-reservoir of intestinal microbiota that re-colonize the lower gastrointestinal tract after during the recovery from infection.

Interpreting the available data form the relevant published studies, it cannot be safely concluded whether a previous appendicectomy can be protective or not from a future CDI, as well as if it could be a factor contributing to a CDI relapse. However, it appears that in patients suffering from CDI, a prior appendicectomy is not associated with the clinical and pathological severity of the infection. It is evident that further observational studies need to be performed in order to provide definitive answers to the above mentioned issues and clarify the role of the appendix in the course of CDI.
